# Late-onset sepsis due to urinary tract infection in very preterm neonates is not uncommon

**DOI:** 10.1007/s00431-017-3030-9

**Published:** 2017-10-23

**Authors:** Alexander B. Mohseny, Veerle van Velze, Sylke J. Steggerda, Vivianne E. H. J. Smits-Wintjens, Vincent Bekker, Enrico Lopriore

**Affiliations:** 1 Department of Neonatology, Willem-Alexander Children’s Hospital, Leiden University Medical Center, Leiden, The Netherlands; 2 Department of Pediatric Infectious Diseases, Willem-Alexander Children’s Hospital, Leiden University Medical Center, Leiden, The Netherlands

**Keywords:** Urinary tract infection, *Candida albicans*, Late-onset sepsis, Premature infant, Central venous catheter

## Abstract

Urinary tract infection (UTI) is a common cause of sepsis in infants. Premature infants hospitalized at a neonatal intensive care unit often have risk factors for infection. In this group, the risk of UTI is not clearly known, and guidelines for urine analysis are not unanimous. We aimed to identify the risk of UTI in premature infants with central lines, suspected of late-onset sepsis. We analyzed all 1402 infants admitted to our hospital between 2006 and 2014 with a gestational age less than 32 weeks. Six hundred sixty-two episodes of sepsis evaluations were found with an unknown source of infection based on clinical symptoms. In half of this group, urine analysis was performed identifying UTI in 11.3% (24/212). In 13 of these infants (54%) with a UTI, infection was due to *Candida albicans*. In at least four episodes, the diagnosis and treatment would have been delayed if urine analysis had not been performed.

*Conclusion*: Based on these findings, we conclude that in premature infants with central lines, urine analysis should be performed routinely when signs of infection occur beyond 72 h after birth. Urine collection should not be delayed and cultures should preferably be performed before the start of the antibiotic treatment.

**Table:** 

**What is known:** • *In preterm infants, the presence of other risk factors for infection might make clinicians reluctant to obtain urine cultures during sepsis evaluation.* • *An internal survey demonstrated that there is no consensus within the NICUs in The Netherlands regarding urine analysis as part of LOS work-up.*
**What is new:** • *The risk of UTI in the NICU population (11.3%) is comparable to term infants; therefore, urine analysis should be performed routinely when LOS is suspected.* • *Candida albicans was the most frequently (54%) detected pathogen causing UTI in this population.*

## Introduction

Children admitted to the neonatal intensive care unit (NICU) are at increased risk of infections. Infection is an important determinant of length of stay, morbidity, and mortality. The risk of urinary tract infection (UTI) is reported 13.6–16.4% in term infants who present with fever or other signs of infection [1–3]. The overall occurrence of UTI is reported higher in preterm infants as compared to term infants [4]. In both groups, the incidence of UTI increases with postnatal age, varying from 1 to 2% in the first 72 h after birth up to 25% later on [4, 5]. Therefore, routine early-onset sepsis (EOS) work-up in infants does not include urine cultures [5]. UTI in preterm infants may be secondary to bacteremia, but it may also start as primary infection in the urinary tract leading to bacteremia [6]. In both scenarios, diagnosing the UTI is of importance since infection may cause kidney damage or may be secondary to a congenital abnormality of the kidney and the urinary tract (CAKUT).

In preterm infants admitted at the NICU, data regarding urine cultures in the evaluation of late-onset sepsis (LOS) are limited [7]. Nevertheless, since the overall risk of UTI rises with decreasing gestational age, low birth weight, and postnatal age above 72 h after birth, urine should be analyzed when LOS is suspected [4, 5, 7, 8]. However, the presence of other risk factors for infection might make clinicians reluctant to obtain urine cultures during the primary sepsis evaluation [9]. The use of central arterial or venous lines in these infants, providing a direct source of bacteremia, is such a risk factor and often the cause of blood stream infections [10]. This might suggest that a central line infection has a higher probability than a primary UTI to cause sepsis in these infants. Furthermore, presumed complications of sterile catheterization or suprapubic aspiration hamper urine collection and alternative collection methods such as bag collection have high rates of contamination [11]. An internal survey demonstrated that half of the NICUs in The Netherlands consistently include urine analysis as part of LOS work-up while the remaining centers only collect urine when additional UTI related symptoms are present. Therefore, rationale and recommendations are needed to either underline or discourage routine urine culturing when premature infants with central lines are evaluated for LOS.

The aim of this study was to evaluate the risk of UTI among premature infants with central lines who were evaluated for LOS. In addition, concordant blood cultures and ultrasonography images were analyzed to investigate the consequences of not (timely) diagnosing the UTI. Recorded data included side effects of sampling urine such as registered pain or complications, the treatment, and the presence of urinary tract malformations.

## Materials and methods

### Study design

We conducted a single center cohort study of all infants with a gestational age < 32 weeks hospitalized at the NICU of the Leiden University Medical Center between 01 and 01-2006 and 01-01-2014 (Table 1). Infants with known congenital anomalies, in particular congenital anomalies of the kidney and urinary tract, were excluded from the study.

**Table 1:** Patients’ characteristics of the study cohort

Patients	*N*	
*Total analyzed*	1402	
*Congenital abnormalities excluded*	86	
Sepsis episodes analyzed	1548 (from 1316 patients)	
*Episodes excluded*	1113	
*Episodes included* ^*iii*^	435 (from 389 patients)	
Gestational age	Range (weeks)	Mean
	23–31	27.7
Birth weight	Range (grams)	Mean
	430–2040	1044
Gender	*N*	Ratio
*Male*	216	1.25:1
*Female*	173	
Central catheters^iv^	*N*	
*Umbilical*	336	
*Peripheral*	222	
Time of sepsis evaluation	Range (hours after birth)	Mean
	73–1781	264
Urine cultures	*N*	Positive (%)
Total	212 (from 192 patients)	11.3
Suprapubic aspiration	18	5.6
Sterile urethral catheterization	102	15.6
Catheter a demeure	30	13.3
Bag collection	56	5.3

Different characteristics are grouped within the emphasized factor, such as patients, urine cultures etc

^i^Congenital abnormalities included syndromes associated with chromosomal anomalies, cyanotic cor vitia, congenital defects of an organ system including the urinary tract, hydrops fetalis, and proven metabolic disorder

^ii^The following sepsis evaluations were excluded:

• Pre/perinatal risk factors (*N* = 743) defined as unknown cause of the premature birth, infants with clinical signs of sepsis at birth, or a combination of two or more factors including maternal fever, maternal group B streptococcal (GBS) positivity without antibiotic prophylaxis or previous child with GBS sepsis, premature rupture of the membranes longer than 18 h before birth or asphyxia.

• No central line within 24 h of LOS evaluation (*N* = 81).

• EOS (*N* = 146).

• Suspected NEC (*N* = 41).

• Suspected VAP (*N* = 42).

• Iatrogenic (*N* = 8) after reposition or manipulation of a central venous line.

• Unknown reason of the sepsis evaluation (*N* = 52).

^iii^Please note that this number of episodes exceeds the total number of infants included, since multiple episodes of sepsis were included when the episodes were unrelated

^iv^Some included infants had umbilical and peripheral central lines

### Data collection

Patient records of included infants were anonymously analyzed to identify any episodes of sepsis evaluation. Demographic details, urine and blood cultures, postnatal age at sepsis work-up (in hours after birth), presence of central lines, therapy of choice and duration, method of urine sampling registered complications of urine sampling, and ultrasonography results were collected for analysis.

### Definitions

LOS was defined as clinical signs of sepsis as judged by the physician, developing more than 72 h after birth [12, 13]. When an infant was evaluated for sepsis more than once, all new unrelated episodes, i.e., free of clinical sings or positive relevant cultures between two episodes, were included as separate events. Urine specimens were obtained by one of four methods: sterile urethral catheterization (SUC), suprapubic aspiration (SA), bag collection (BC), or collecting fresh urine from a catheter a demeure (CAD). Urine cultures with mono-cultural bacterial growth were considered positive based on the number of colony-forming units per milliliter (CFU/ml) dependent of the method of collection [14]. Central lines included umbilical arterial or venous lines and peripheral inserted central venous lines.

### Sepsis work-up and urine culture

Sepsis work-up always included a blood culture prior to the start of antibiotic treatment. Culture of other specimens including urine and cerebral spinal fluid was advised but left to the discretion of the attending physician. Any bacterial growth from urine collected by SA was considered positive. Mono-cultural bacterial growth of > 10.000–100.000 CFU/ml collected by SUC or freshly collected CAD samples was defined as positive. Upon pathogenic growth from CAD urine, the catheter was removed and antibiotic treatment was initiated. Follow-up urine analysis was performed to control the efficacy of the treatment. Urine specimens from BC were only considered positive if there was mono-cultural growth > 100.000 CFU/ml of a urinary pathogen and the clinical course supported UTI.

### Statistical analysis

The minimal needed size of the study population was based on the identification of UTI risk in preterm NICU-hospitalized infants with central lines. Reported data suggested UTI rates around 10% in infants without central lines [1, 2, 4], and we defined that a risk rate of 2% or higher (since a risk of 1–2% is accepted in EOS) would justify routine urine analysis as part of LOS evaluation. The power calculation showed that at least 360 patients should be included to detect a difference rate of 2 to 10% with a power (beta) of 0.9 allowing an error rate (alpha) of 0.05. Data was analyzed by using IBM SPSS data analysis software version 22.

## Results

### Cohort population

Between 2006 and 2014, 1402 newborns with a gestational age less than 32 weeks were admitted to our unit of which 1316 were included. Together, these infants had undergone 1548 episodes of unrelated sepsis evaluation. Next, all episodes with sepsis evaluation because of pre/perinatal infection risks or with a known or plausible origin outside the urinary tract such as necrotizing enterocolitis (NEC) or ventilator-associated pneumonia (VAP) were excluded, resulting into 662 remaining episodes with sepsis of unknown origin. Of these episodes, 581 had central lines at the time of sepsis work-up, and 435 (from 389 neonates) were suspected of infection beyond 72 h after birth. In this group, urine was analyzed in 212 episodes (from 192 neonates, 49%, Table 1).

### Number of UTI

Out of 212 urine cultures, a total of 24 (11%) showed pathogenic growth. Thirteen times (54%) *Candida albicans* was cultured, three times (13%) *Escherichia coli*, three times (13%) *Staphylococcus aureus*, one (4%) *Morganella morganii*, one *Klebsiella pneumoniae*, one *Enterobacter cloacae*, one *Klebsiella oxytoca* , and one *Streptococcus agalactiae* (Table 2).

**Table 2:** Overview of the pathogens that were cultured from urine analysis and related characteristics

Gestational age (weeks)	Time of evaluation (hours after birth)	Pathogen	Urine collection method	Ultrasonography of the urinary tract	Concordant growth in other cultures
23	1200	Candida albicans	BC	Normal	Skin
24	137	Candida albicans	SUC	Not performed	Skin and blood
24	239	Candida albicans	SUC	Normal	Blood
24	279	Candida albicans	SA	Normal	Skin and blood
25	133	Candida albicans	BC	Not performed	Skin
26	172	Candida albicans	SUC	Normal	Pharynx
26	88	Candida albicans	SUC	Normal	Blood
26	82	Candida albicans	SUC	Fungus balls	Blood and CSF
26	344	Candida albicans	SUC	Normal	Sputum and blood
26	301	Candida albicans	SUC	Normal	Blood
27	416	Escherchia coli	CAD	Not performed	Eye fluid
27	864	Klebsiella oxytoca	SUC	Normal	No
27	278	Candida albicans	SUC	Normal	No
28	281	Candida albicans	SUC	Normal	No
28	840	*Klebsiella pneumoniae*	BC	Not performed	No
28	194	Escherchia coli	SUC	Not performed	No
28	125	*Staphylococcus aureus*	SUC	Not performed	Blood
29	651	*Enterobacter cloacae*	CAD	Not performed	Sputum
29	627	Escherchia coli	CAD	Normal	No
29	195	Staphylococcus aureus	CAD	Not performed	Blood
29	272	Candida albicans	SUC	Normal	No
29	452	*Streptococcus agalactiae*	SUC	Not performed	Blood
30	310	Morganella morganii	SUC	Normal	Blood
30	93	Staphylococcus aureus	SUC	Not performed	Blood

*CSF* cerebrospinal fluid, *SUC* sterile urethral catheterization, *SA* suprapubic aspiration, *BC* bag collection, *CAD* catheter a demeure (CAD)

### Method of urine collection

Urine was collected from 102 infants by SUC (48%), 56 times by BC (26%), CAD in 30 episodes (14%), and SA in 18 episodes (9%). Of the six remaining episodes (3%), specifics about the method of collection could not be tracked. Side effects such as pain or complications after urine collection were not reported. From the 24 positive urine cultures, 16 were collected by SUC (67%), 4 by CAD (17%), 3 by BC (12%), and 1 by SA (4%).

### Ultrasonography

To detect any (possibly missed) urinary tract abnormalities related to UTI, reports of all performed ultrasonography examination were analyzed. Within the total group of 1402 infants, ultrasonography of the urinary tract was performed 138 times (9%). In 20 of these studies, abnormalities were found (14%). These 20 abnormal outcomes were originating from 16 infants; as in some infants, imaging of the urinary tract was repeated for follow-up. The abnormalities included abnormal size for the age (3), hydronephrosis unrelated to infection (7), nephrocalcinosis (1), renal vein thrombosis (1), extra renal pyelum (2), and fungus balls in two cases. No abnormalities predisposing for UTI were found and only the two cases with fungus balls were interpreted as complications of UTI. Within the group with positive pathogen growth from the urine culture (24 episodes), 14 ultrasound examinations were performed and showed an abnormality in only one infant; renal pelvis fungus balls in both kidneys. So, one infant was found with reported fungus balls in one kidney without pathogenic growth from urine and blood cultures. This infant was referred to our NICU after *Candida* UTI was diagnosed, and systemic treatment was initiated elsewhere explaining the negative cultures at our NICU.

## Discussion

This study was designed to investigate the risk of UTI in NICU-admitted premature infants (gestational age < 32 weeks) with central lines who were suspected of LOS. We found a high risk (11.3%) of UTI in this specific population. Our data show that UTI is not uncommon and that the source of infection in 1 out of 10 episodes is the urinary tract. Interestingly, in these neonates, only in 40% urine was cultured. Prior to this study, we hypothesized that this was related to the assumption of clinicians that UTI is rare in this population because the presence of central lines is a more likely sepsis risk factor. Moreover, lack of a rigid and evidence-based protocol for this group of infants, such as available protocols for term neonates [14], might explain the motivation of the clinician not to prioritize urine analysis.

In addition, urine collection in premature infants by catheterization or suprapubic aspiration might be challenging. To overcome this, often, bag collection was used to screen the urine within the first sepsis evaluation and often repeated by an alternative method for a clean catch to culture when the screening indicated possible infection or contamination. However, repeating the collection results in a delay during which the patient most often has received antibiotics, which might cause false negative urine cultures. Alternative methods such as bladder stimulation and paravertebral lumbar massage techniques are reported with high rates of contamination [11, 15]. In this cohort, UTI was found in three infants by using the method of bag collection showing mono-cultural growth without any sings of contamination. Although the possibility of contamination is not excluded, only three out of 56 BC urine cultures (5%) were considered positive. Moreover, based on these positive cultures, infants were treated with systemic therapy, and following urine cultures after treatment were sterile.

UTI was found in 24 infants, more than half (54%, *n* = 13) caused by *Candida albicans* who received systemic treatment. Candidiasis is known as a frequent cause of infection in NICU-admitted patients [16–19] and is related with a high mortality. From the 13 infants suffering from *Candida albicans* infection, most also had positive *Candida* growth from other cultures such as blood and skin. However, in six episodes, *Candida* was not detected in blood cultures, and concordant growth in skin and/or saliva cultures was only detected in three episodes, indicating that the *Candida* infection might have been missed, and the treatment might have been delayed if the urine cultures were not performed. So, in these patients, urine analysis was of crucial importance for their treatment and prognosis.

During the study period, fluconazole prophylaxis to reduce the risk of *Candida* infection was not routinely used at our NICU. Since the introduction of routine fluconazole prophylaxis in 2013, in premature infants with a gestational age < 27 weeks and/or birth weight < 750 g, only one case of *Candida* UTI was detected (positive urine cultures obtained by SUC). This might indicate that strict fluconazole prophylaxis might result in less UTI due to candida infections. However, urine analysis as part of LOS evaluation remains of crucial importance (in this case blood and surface cultures showed no pathogenic growth). Prospective studies in cohorts with fluconazole prophylaxis are needed to investigate the frequency of UTI in this population.

By conducting this cohort study, we conclude the following:

1. The risk of UTI in the studied population (11.3%) is comparable to term infants, this might guide clinical treatment and have implications for follow-up.
2. Urine analysis should, therefore, be performed routinely when LOS is suspected in preterm neonates, also in the presence of central lines as the presumed risk factor for infection.
3. There is no evidence for complications caused by urine collection independent of the method used, so collecting urine should not be delayed and preferably performed before the start of antibiotic treatment.
4. Prospective studies are needed to determine the incidence of UTI in the NICU population without UTI predisposing factors when fluconazole prophylaxis is administered to examine whether routine urine analysis would still be justified.

## Abbreviations

BC: Bag collection
CAD: Catheter a demeure
EOS: Early-onset sepsis
LOS: Late-onset sepsis
NEC: Necrotizing enterocolitis
NICU: Neonatal intensive care unit
SA: Suprapubic aspiration
SUC: Sterile urethral catheterization
UTI: Urinary tract infection
VAP: Ventilator-associated pneumonia
